# PAI1 regulating CHRNA1 contributes to primary focal hyperhidrosis: Clinical and experimental studies

**DOI:** 10.1016/j.omtn.2025.102566

**Published:** 2025-05-16

**Authors:** Ru-Jie Zheng, Nan-Long Lin, Meng-Long Zhang, Rui-Qin Qiu, Feng-Qiang Yu, Xu Li, Jian-Bo Lin

**Affiliations:** 1 Department of Anesthesiology, First Affiliated Hospital of Fujian Medical University, Fuzhou 350005, China; 2 Department of Anesthesiology, National Regional Medical Center, Binhai Campus of the First Affiliated Hospital, Fuzhou 350005, China; 3 Anesthesiology Research Institute, the First Affiliated Hospital, Fujian Medical University, Fuzhou 350005, China; 4 Department of Thoracic Surgery, First Affiliated Hospital of Fujian Medical University, Fuzhou 350005, China; 5 Department of Thoracic Surgery, National Regional Medical Center, Binhai Campus of the First Affiliated Hospital, Fujian Medical University, Fuzhou 350212, China; 6 Fujian Medical University, Fuzhou, China; 7 Fujian Key Laboratory of Precision Medicine for Cancer, The First Affiliated Hospital, Fujian Medical University, Fuzhou 350005, China

**Keywords:** MT: Oligonucleotides: Diagnostics and Biosensors, primary focal hyperhidrosis, nicotinic acetylcholine receptor alpha 1 subunit, plasminogen activator inhibitor-1, sweet secretion

## Abstract

Primary focal hyperhidrosis (PFH) is a debilitating condition characterized by localized excessive sweating, yet its underlying mechanisms remain poorly understood. In this study, sweat gland tissues from PFH patients (*n* = 204) and healthy controls (*n* = 60) were analyzed to assess the mRNA and protein levels of plasminogen activator inhibitor 1 (PAI-1) and nicotinic acetylcholine receptor alpha 1 subunit (CHRNA1) using RT-qPCR and western blotting. Primary sweat gland cells were isolated for *in vitro* experiments, and a pilocarpine-induced hyperhidrosis mouse model was established to evaluate the therapeutic effect of recombinant human PAI-1 (rhPAI-1). PFH patients showed significantly reduced PAI-1 expression and elevated CHRNA1 expression compared to controls (*p* < 0.01). Treatment with rhPAI-1 downregulated CHRNA1 and aquaporin 5 (AQP5) expression in sweat gland cells and decreased sweat secretion and serum acetylcholine levels *in vivo*. These results suggest that PAI-1 negatively regulates CHRNA1 and AQP5 expression, offering new insights into the molecular pathology of PFH and identifying PAI-1 as a potential therapeutic target for hyperhidrosis.

## Graphical abstract

**Figure:**
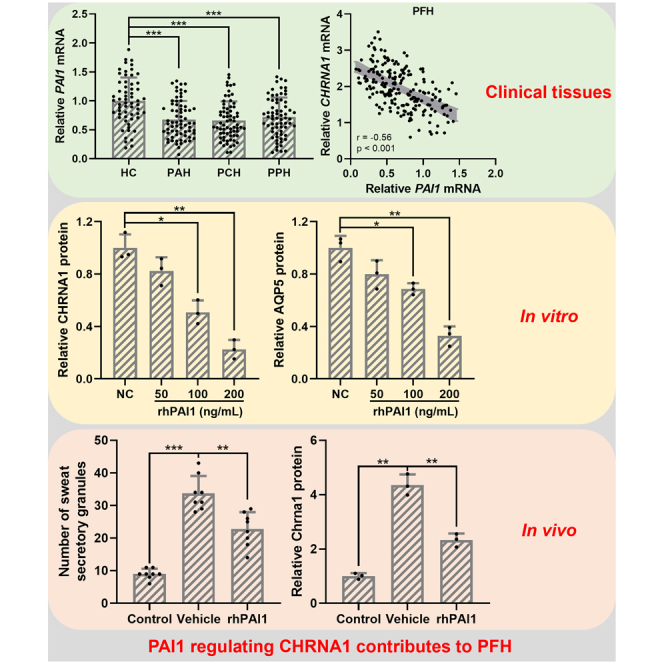

## Teaser

PFH patients show significantly reduced PAI-1 expression and elevated CHRNA1 expression compared to controls. Treatment with rhPAI-1 downregulates CHRNA1 and aquaporin 5 expression in sweat gland cells and decreases sweat secretion and serum acetylcholine levels *in vivo*.

## Introduction

Primary focal hyperhidrosis (PFH) is a perplexing medical condition characterized by excessive sweating localized to specific regions of the body, notably the palms, soles, axillae, and face, in the absence of any underlying systemic cause.^1^^,^^2^ This condition poses significant challenges to affected individuals.^3^^,^^4^ The constant and unpredictable nature of excessive sweating can lead to embarrassment, social withdrawal, and decreased self-esteem.^5^^,^^6^ Despite its prevalence and the substantial burden it imposes, PFH remains underrecognized and undertreated in clinical practice.^7^ This may partly stem from misconceptions about sweating as a benign physiological process and a lack of awareness among both healthcare providers and the general public regarding the distressing nature of PFH.^8^ Additionally, the absence of visible signs or biomarkers complicates diagnosis and may contribute to diagnostic delays or misdiagnosis.^9^

The nicotinic acetylcholine receptor alpha 1 subunit (CHRNA1) gene, encoding the α1 subunit of the nicotinic acetylcholine receptor (nAChR), stands as a pivotal player in the intricate network governing neuromuscular transmission.^10^^,^^11^ Dysregulation of CHRNA1 expression has been implicated in various neuromuscular disorders. Previous studies, including our own, have demonstrated that CHRNA1 expression levels in thoracic sympathetic ganglia and sweat gland tissues of PFH patients are significantly higher compared to healthy individuals.^9^^,^^12^ Inhibition of CHRNA1 has been shown to reduce acetylcholine (ACh) levels—a biomarker of PFH—and downregulate key proteins involved in sweat secretion, such as aquaporin 5 (AQP5) and the calcium channel protein CACNA1C, ultimately suppressing PFH development in mouse models.^13^^,^^14^^,^^15^ These findings highlight CHRNA1 as a potential therapeutic target, although the upstream regulatory mechanisms controlling CHRNA1 remain to be fully elucidated. Understanding these mechanisms is crucial for developing new targeted therapies for PFH.

Plasminogen activator inhibitor 1 (PAI-1) occupies a central position in the intricate network governing fibrinolysis, hemostasis, and extracellular matrix remodeling.^16^^,^^17^ Its pivotal role in modulating the balance between clot formation and dissolution underscores its significance in both physiological processes and a myriad of pathological conditions.^18^ Both PAI-1 and CHRNA1 have been implicated in neuromuscular disorders, albeit through different mechanisms.^19^ Studies have shown that PAI-1 inhibitors can up-regulate the expression level of CHRNA1, suggesting that PAI-1 may play an inhibitory role in the up-regulation of CHRNA1 in PFH.^14^

Due to the roles of PAI-1 in inflammation and tissue remodeling, as well as the critical role of CHRNA1 in neuromuscular transmission, their interaction is significant in the pathophysiological processes of PFH.^14^ Our previous study primarily focused on basic research using mouse models, in which we investigated the role of PAI-1 in PFH pathogenesis through genetic manipulation methods, including overexpression and knockout of PAI-1.^14^ Although this work provided important mechanistic insights, it was limited to animal models and genetic-level interventions without clinical validation.^14^ In contrast, the current study expands upon our earlier findings in two major aspects: first, by analyzing sweat gland tissues from human PFH patients to verify the correlation between PAI-1 and CHRNA1 expression in clinical samples and second, by using recombinant human PAI-1 protein (rhPAI-1) to perform protein-level interventions in both primary sweat gland cells and a pilocarpine-induced mouse model. This approach more closely simulates potential clinical therapeutic strategies, thereby enhancing the translational significance of the study.

Therefore, this study aims to analyze the correlation between PAI-1 and CHRNA1 through clinical tissue sample analysis, building upon the findings from animal experiments. Additionally, we sought to validate the regulatory effect of PAI-1 on CHRNA1 at the protein level using patient-derived primary sweat gland cells and animal models, providing a stronger foundation for future therapeutic exploration in PFH.

## Results

### RNA levels of PAI1 and CHRNA1 in PFH patients

**Figure 1:**
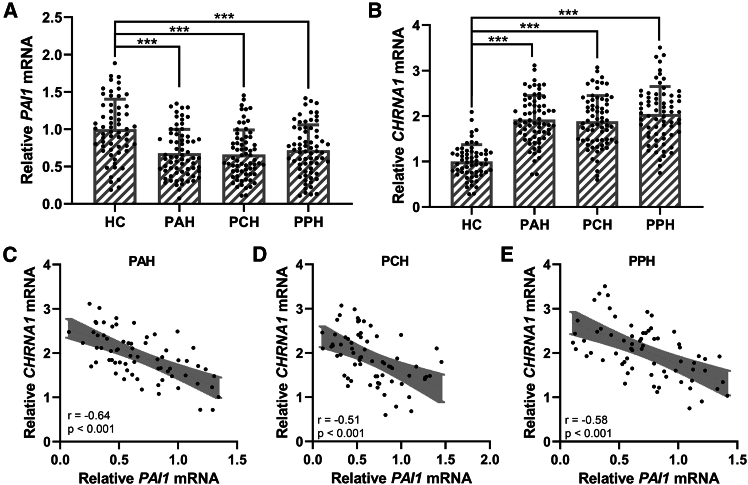
Expressions of PAI1 and CHRNA1 in sweat gland tissues of primary focal hyperhidrosis patients The mRNA levels of PAI1 (A) and CHRNA1 (B) in sweat gland tissues from healthy controls (HCs, *n* = 60, corresponding to the dots), primary axillary hyperhidrosis patients (PAH, *n* = 68, corresponding to the dots), primary craniofacial hyperhidrosis patients (PCH, *n* = 68, corresponding to the dots) and primary palmar hyperhidrosis patients (PPH, *n* = 68) were measured by quantitative real-time PCR. The data were presented with mean ± SD. ∗∗∗*p* < 0.001 from Brown-Forsythe ANOVA test followed by Dunnett’s T3 multiple comparisons test. Pearson correlation analysis of the mRNA levels of PAI1 and CHRNA1 in sweat gland tissues from PAH (C), PCH (D), and PPH (E).

Quantitative real-time PCR (real-time qPCR) was performed to analyze the mRNA levels of PAI-1 and CHRNA1 in sweat gland tissues from healthy controls (HCs, *n* = 60), primary axillary hyperhidrosis (PAH, *n* = 68), primary craniofacial hyperhidrosis (PCH, *n* = 68), and primary palmar hyperhidrosis (PPH, *n* = 68) (Figure 1). Compared to HCs, PFH patients, including PAH, PCH, and PPH patients, exhibited a significant reduction in PAI-1 mRNA expression and a significant increase in CHRNA1 mRNA expression (all *p* < 0.001; Figures 1A and 1B). Pearson correlation analysis further demonstrated a strong negative correlation between PAI-1 and CHRNA1 mRNA levels in the PAH, PCH, and PPH subgroups (*p* < 0.001; Figures 1C–1E).

### The protein expression of PAI1 and CHRNA1 in PFH patients

**Figure 2:**
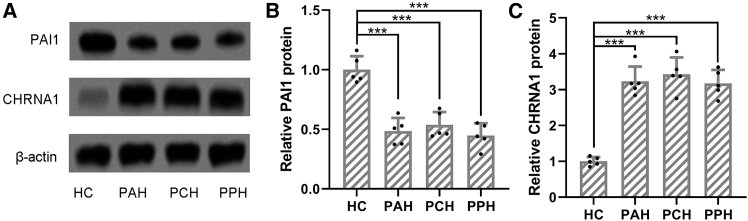
Protein expressions of PAI1 and CHRNA1 in sweat gland tissues of primary focal hyperhidrosis patients The protein levels of PAI1 and CHRNA1 in sweat gland tissues from healthy controls (HCs, *n* = 5 individuals randomly selected from this group), primary axillary hyperhidrosis patients (PAH, *n* = 5 individuals randomly selected from this group), primary craniofacial hyperhidrosis patients (PCH, *n* = 5 individuals randomly selected from this group), and primary palmar hyperhidrosis patients (PPH, *n* = 5 individuals randomly selected from this group) were measured by western blotting (A). β-actin was used as a loading control, and the expressions were normalized to HC (B and C). The data were presented with mean ± SD. ∗∗∗*p* < 0.001 from Brown-Forsythe ANOVA test followed by Dunnett’s T3 multiple comparisons test.

Western blot analysis was conducted on sweat gland tissues from HC (*n* = 5), PAH (*n* = 5), PCH (*n* = 5), and PPH (*n* = 5) cohorts to assess the protein levels of PAI-1 and CHRNA1 (Figure 2). β-actin was used as a loading control. Compared to HCs, PFH tissues (PAH, PCH, and PPH) exhibited a significant decrease in PAI-1 protein expression and a significant increase in CHRNA1 protein expression (all *p* < 0.001; Figures 2A–2C).

### Expressions of PAI1 and CHRNA1 in primary sweat gland cells

**Figure 3:**
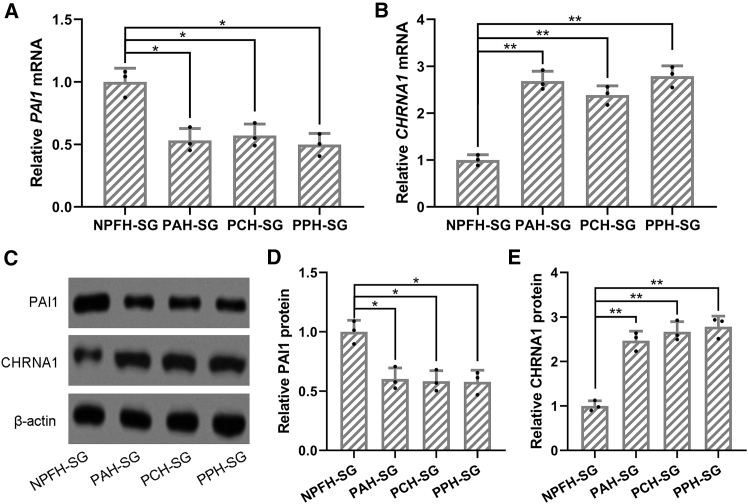
Expressions of PAI1 and CHRNA1 in primary sweat gland cells from primary focal hyperhidrosis patients The mRNA levels of PAI1 (A) and CHRNA1 (B) in primary sweat gland cells from HC, PAH, PCH, and PPH were measured by RT-qPCR. The protein levels of PAI1 and CHRNA1 in primary sweat gland cells from HC, PAH, PCH, and PPH were measured by western blotting (C). β-actin was used as a loading control, and the expressions were normalized to NPFH-SG (D and E). The cell homogenates were used for three repeats. ∗*p* < 0.05 and ∗∗*p* < 0.01 from Brown-Forsythe ANOVA test followed by Dunnett’s T3 multiple comparisons test.

Primary sweat gland cells were isolated from PFH and non-PFH (NPFH) individuals, and the expression levels of PAI-1 and CHRNA1 were evaluated (Figure 3). Real-time qPCR analysis showed significantly lower PAI-1 mRNA levels (all *p* < 0.05) and higher CHRNA1 mRNA levels (all *p* < 0.01) in PFH-derived cells compared to NPFH controls (Figures 3A and 3B). Consistently, western blot results confirmed decreased PAI-1 (all *p* < 0.01) and increased CHRNA1 protein expression in PFH sweat gland cells relative to NPFH-SG cells (Figures 3C–3E).

### Impact of PAI1 on the protein expressions of CHRNA1 and AQP5

**Figure 4:**
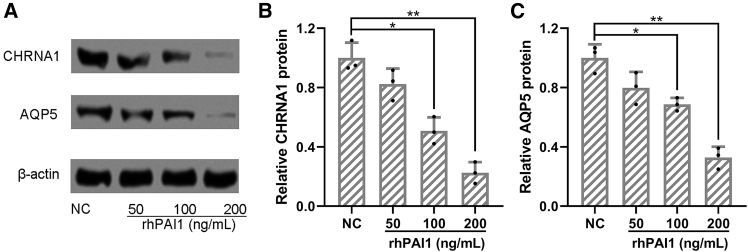
Recombinant human PAI1 inhibited protein expressions of CHRNA1 and AQP5 in primary sweat gland cells from primary focal hyperhidrosis patients Different concentrations of recombinant human PAI1 (rhPAI1, 50, 100, 200 ng/mL) were added into the culture of primary sweat gland cells from primary focal hyperhidrosis patients for 48 h. The proteins levels of CHRNA1 and AQP5 were measured by western blotting (A). β-actin was used as a loading control, and the expressions were normalized to NC (B and C). The cell homogenates were used for three repeats. ∗*p* < 0.05 and ∗∗*p* < 0.01 from Brown-Forsythe ANOVA test followed by Dunnett’s T3 multiple comparisons test.

As shown in Figure 4, we investigated the effects of recombinant human PAI-1 (rhPAI-1) on the protein expression levels of CHRNA1 and AQP5 in primary sweat gland cells derived from PFH patients. Based on a previously published study^20^ and our preliminary optimization experiments, cells were treated with rhPAI-1 at concentrations of 50, 100, and 200 ng/mL for 48 h, conditions determined to be effective without inducing cytotoxicity. Western blot analysis revealed that rhPAI-1 treatment significantly reduced the protein expressions of both CHRNA1 and AQP5 in a concentration-dependent manner compared to untreated controls (Figures 4A–4C). These results suggest that PAI-1 exerts a direct regulatory effect on key molecular components of sweat gland function, potentially contributing to the modulation of hyperhidrosis pathology.

### Effect of rhPAI1 on sweat secretion in hyperhidrosis mice

**Figure 5:**
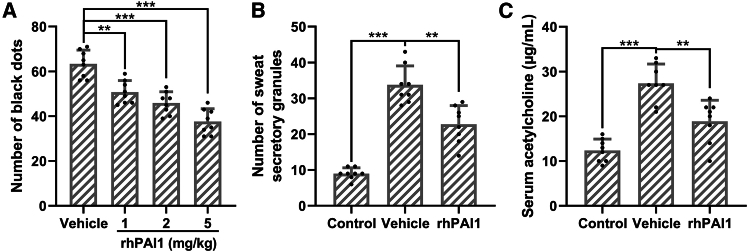
Recombinant human PAI1 attenuated the sweat secretion in hyperhidrosis mice Mice were administered with vehicle and recombinant human PAI1 for 1 week before the induction of hyperhidrosis. The number of black dots were calculated (A). Then the dose of 5 mg/kg was used. The number of sweat secretory granules were counted (B), and the concentration of acetylcholine in serum was detected by ELISA (C). The data were presented with mean ± SD. *n* = 8 for each group. ∗∗*p* < 0.01 and ∗∗∗*p* < 0.001 from Brown-Forsythe ANOVA test followed by Dunnett’s T3 multiple comparisons test.

To evaluate the effect of rhPAI-1 on sweat secretion, hyperhidrosis was induced in mice by intraperitoneal injection of pilocarpine hydrochloride (5 mg/kg body weight). Prior to hyperhidrosis induction, mice were pretreated with either vehicle or rhPAI-1 for 1 week. Sweat secretion was quantified by counting the number of black sweat dots on the paws after 5 min of pilocarpine stimulation (Figure 5A). Pretreatment with rhPAI-1 at doses of 1, 2, and 5 mg/kg via tail vein injection demonstrated a dose-dependent reduction in sweat secretion, with 5 mg/kg being the most effective dose. Further analysis showed that rhPAI-1 administration at 5 mg/kg significantly reduced the number of sweat secretory granules in sweat glands (*p* < 0.01; Figures S1 and 5B) and decreased serum acetylcholine levels as determined by ELISA (*p* < 0.01; Figure 5C). These findings indicate that rhPAI-1 ameliorates hyperhidrosis symptoms in mice by reducing both sweat gland activity and systemic acetylcholine concentrations.

### The impact of rhPAI1 on the expression of Chrna1 and Aqp5 in the sweat glands of hyperhidrosis mice

**Figure 6:**
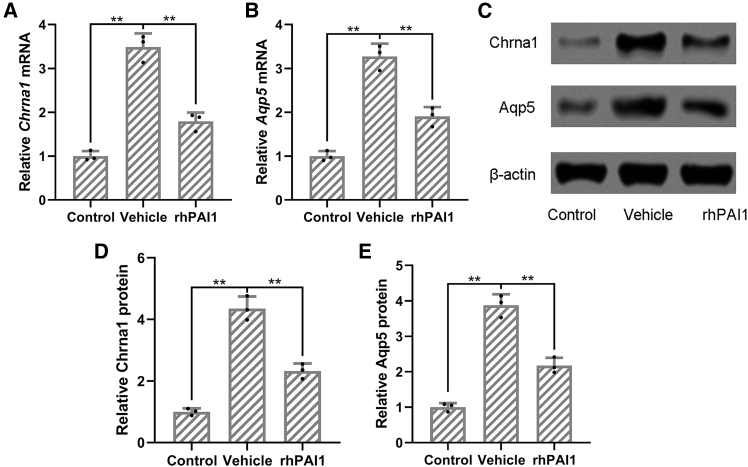
Recombinant human PAI1 inhibited the expressions of Chrna1 and Aqp5 in the sweat gland of hyperhidrosis mice Mice were administered with vehicle, 5 mg/kg recombinant human PAI1 for 1 week before the induction of hyperhidrosis. RT-qPCR was used to detect the mRNA expression of Chrna1 and Aqp5 in the sweat gland of hyperhidrosis mice (A and B). Western blotting was used to detect the protein expression of Chrna1 and Aqp5 in the sweat gland of hyperhidrosis mice (C). β-actin was used as a loading control, and the expressions were normalized to control (D and E). The data were presented with mean ± SD. *n* = 3 for each group. ∗∗*p* < 0.01 from Brown-Forsythe ANOVA test followed by Dunnett’s T3 multiple comparisons test.

To investigate the molecular mechanisms underlying the effects of rhPAI-1, mRNA and protein levels of Chrna1 and Aqp5 in sweat gland tissues were assessed. Mice pretreated with rhPAI-1 (5 mg/kg) or vehicle were analyzed using real-time qPCR and western blotting (Figure 6). β-actin served as the internal control for normalization. Real-time qPCR results demonstrated that rhPAI-1 treatment significantly decreased the mRNA expression levels of both Chrna1 and Aqp5 compared to the vehicle group (*p* < 0.01; Figures 6A and 6B). Western blot analysis further confirmed a corresponding reduction at the protein level (*p* < 0.05; Figures 6C–6E). These results suggest that rhPAI-1 suppresses the expression of key molecular targets involved in sweat gland function, supporting its potential as a therapeutic agent for hyperhidrosis.

## Discussion

In this study, we demonstrated that PAI-1 expression was significantly decreased, whereas CHRNA1 expression was increased in sweat gland tissues from patients with PFH compared to HCs. Using both clinical tissue samples and experimental models, we confirmed that rhPAI-1 treatment effectively downregulated CHRNA1 and AQP5 expression at both the mRNA and protein levels. Functionally, rhPAI-1 administration reduced sweat secretion and serum acetylcholine levels in a pilocarpine-induced hyperhidrosis mouse model, supporting the therapeutic potential of targeting the PAI-1/CHRNA1 pathway in PFH.

One of the strengths of this study is the integration of clinical and experimental approaches, including molecular analyses of patient-derived tissues and validation in primary sweat gland cells and animal models. By examining both mRNA and protein expression levels, we provided multi-level evidence supporting the regulatory role of PAI-1 on CHRNA1 and its involvement in sweat gland function. Moreover, the protein-level intervention strategy using rhPAI-1 more closely mimics potential clinical therapeutic approaches compared to previous studies focusing on genetic manipulation. Previous research, including our earlier study, primarily utilized genetic models such as PAI-1 overexpression or knockout in mice to elucidate the mechanistic role.^21^^,^^22^^,^^23^ Although these studies provided important mechanistic insights, they were limited by their reliance on artificial genetic modifications, which may not fully represent therapeutic realities in clinical settings. In contrast, the current study employed recombinant human PAI-1 as a protein-based therapeutic agent, allowing us to simulate a more clinically translatable intervention and evaluate its effects both *in vitro* and *in vivo*.

Our study unveils novel insights into the pathophysiology of PFH by elucidating the regulatory role of PAI-1 on the expression of the CHRNA1. Through a comprehensive analysis of clinical tissue samples and experimental models, we demonstrate that PAI-1 inhibition leads to downregulation of CHRNA1 expression, consequently attenuating sweat secretion in PFH. This mechanistic understanding highlights PAI-1 as a promising therapeutic target for PFH management, offering a novel approach to alleviate symptoms and improve patients' quality of life.

Despite providing valuable insights into the molecular mechanisms of PFH and identifying potential therapeutic targets, our study has several limitations. First, the sample size in both clinical tissue sample analysis and experimental studies was relatively small, potentially limiting the generalizability of the findings. Moreover, our focus on CHRNA1 and its downstream effects may overlook other important molecular pathways contributing to PFH. Additionally, although animal models offer valuable insights, they may not fully capture the complexity of human PFH. Furthermore, the short-term follow-up in our experimental studies may not adequately assess the long-term effects and potential side effects of interventions targeting PAI-1 and CHRNA1. Moreover, although we identified CHRNA1 as a potential therapeutic target, our study did not fully elucidate the underlying molecular mechanisms governing its regulation of PFH progression.

Future research should aim to further dissect the molecular mechanisms linking PAI-1 to CHRNA1 regulation, explore potential off-target effects of PAI-1 modulation, and evaluate the efficacy of rhPAI-1 or related agents in clinical trials. These efforts will contribute to the development of safer, more effective, and durable therapeutic strategies for PFH.

### Conclusions

In this study, we demonstrated that PAI-1 expression is negatively correlated with CHRNA1 expression in sweat gland tissues from patients with PFH. Using both clinical samples and experimental models, we further confirmed that rhPAI-1 intervention downregulated CHRNA1 expression and ameliorated the hyperhidrosis phenotype, suggesting a critical regulatory role of PAI-1 in PFH pathogenesis. The major strengths of our study include the integration of clinical validation with mechanistic exploration, and the use of protein-level intervention strategies, which enhances the translational relevance of our findings. This work identifies the PAI-1/CHRNA1 axis as a potential novel therapeutic target for PFH. Future studies are needed to validate these findings in larger, multicenter patient cohorts and to explore the *in vivo* therapeutic potential of modulating the PAI-1/CHRNA1 pathway. Further investigations into additional molecular pathways involved in PFH could also provide a broader foundation for developing targeted, long-term treatment strategies to improve patients’ quality of life.

## Materials and Methods

### Gland collection

Sweat gland tissue samples were collected from a total of 264 individuals, including 68 cases each of PAH, PPH, and PCH, along with 60 HCs. All participants were enrolled at the First Affiliated Hospital of Fujian Medical University under approved protocols (#2023-511).

For patients with PFH, sweat gland tissues were obtained during surgical procedures (single-hole thoracoscopic sympathectomy or axillary surgery).^10^ Tissue samples approximately 2 mm in width and 5 mm in length were harvested from the right axillary incision, including full-thickness skin containing sweat glands. In selected cases, similar volume tissue samples were collected from the contralateral (non-affected) side as intra-individual controls when applicable.

HC tissues were collected from individuals without any history of palmar or axillary hyperhidrosis, familial predisposition, or axillary osmidrosis. This sample design allowed for a comprehensive evaluation of PAI-1 and CHRNA1 expression across different PFH subtypes.

### Real-time qPCR

Total RNAs were isolated from tissues using Trizol reagent, followed by reverse transcription into cDNA. Real-time PCR was conducted using SYBR green agent on a thermocycler. The study analyzed four groups, including 60 HCs and 68 cases each of PAH, PPH, and PCH. Gene expressions of PAI-1 and CHRNA1 were examined. Specific primer sequences were used for human and mouse samples. For human samples:

PAI1: F: 5′ACCGCAACGTGGTTTTCTCA3′,
R: 5′TTGAATCCCATAGCTGCTTGAAT3′
CHRNA1: F: 5′TCATCATTCCCTGCCTGCTCTTCT3′,
R: 5′TCTCTGCAATGTACTTCACGCCCT3′
GAPDH: F: 5′GCACAGTCAAGGCCGAGAAT3′,
R: 5′GCCTTCTCCATGGTGGTGAA3′

For mouse samples:

Aqp5: F: 5′AGAAGGAGTGTGTTCAGTTGC3′,
R: 5′GCCAGAGTATGGCCGGAT3’
Chrna1: F: 5′ TCATCATTCCCTGCCTGCTCTTCT3′,
R: 5′TCTCTGCAATGTACTTCACGCCCT3’
Gapdh: F: 5′- AGGTGGTGTGAACGGATTTG-3′,
R: 5′- TGTAGACCATGAGTTGAGGTCA-3′

### Western bolt

Five tissue samples from each of the four groups were randomly selected for western blot experiments. Sweat gland tissue (0.2 g) was pulverized using liquid nitrogen, and proteins were extracted using Ripa lysate. Primary antibodies: PAI1 (Abcam, cat#: ab222754) and CHRNA1 (Abcam, cat#: ab308306).

### Cell isolation and culture

Primary sweat gland cells were isolated from axillary sweat gland tissues obtained from patients diagnosed with PFH and from non-PFH individuals. The isolation procedure was based on our established protocol and prior studies. Briefly, sweat gland tissues were minced into small blocks and subjected to enzymatic digestion using 0.2% collagenase type I at 37°C for 2 h. Following digestion, the tissue suspensions were filtered through a 70-μm cell strainer, centrifuged, and the cell pellets were resuspended in DMEM/F-12 medium supplemented with 10% fetal bovine serum, 1% penicillin-streptomycin, and 10 ng/mL rhEGF. Cells were cultured in a humidified incubator at 37°C with 5% CO_2_, and the adherent epithelial cell populations were maintained for further experiments. The identity of the isolated sweat gland cells was confirmed by immunostaining for cytokeratin 7, a known marker of eccrine sweat gland epithelial cells. Sweat gland cells derived from PFH patients were designated as PFH-SG, whereas cells derived from non-PFH individuals were designated as NPFH-SG. For specific experiments involving gene expression modulation, cells were transfected with the acvr1 vector upon reaching approximately 70% confluence.

### Animals

A hyperhidrosis mouse model was established through intraperitoneal injection of pilocarpine hydrochloride (5 mg/kg body weight), followed by a 5-min stimulation period to induce perspiration, with subsequent measurement of sweat secretion via photography of black spots on the paws. Prior to model construction, mice were subjected to 1 week of treatment with varying doses (1, 2, and 5 mg/kg) of recombinant human PAI-1 protein (ab92774, Abcam), administered via tail vein injection once daily. General mouse rearing conditions included 12-h light-dark cycle, temperature maintenance at 22°C–24°C, relative humidity of 50%, ad libitum access to standard laboratory chow and water, provision of bedding material for nesting and environmental enrichment in cages, and regular health monitoring and veterinary care. Animal studies were approved by Fujian Medical University (#IACUC FJMU 2023-Y-0601).

### Statistical analysis

Data were presented as mean ± standard deviation (SD) and analyzed using GraphPad Prism 7 software. Significant differences were assessed using Brown-Forsythe ANOVA test followed by Dunnett’s T3 multiple comparisons test, with a significance level set at *p* < 0.05.

## Data availability

The data could be obtained upon reasonable request to the corresponding author.

## Acknowledgments

This study was supported by Joint Funds for the Innovation of Science and Technology, Fujian Province (2023Y9093; 2024Y9120), Medical Innovation Project of Fujian Province (Grant 2024CXB004), and Fujian Provincial Finance Project (BPB-2024LJB).

## Author contributions

R.-J.Z., N.-L.L., M.-L.Z., R.-Q.Q., F.-Q.Y., X.L., and J.-B.L. conducted the experiments, analyzed the data, and wrote the original manuscript. J.-B.L. conceived and supervised the study. R.-J.Z. and J.-B.L. revised the manuscript.

## Declaration of interests

The authors declare that they have no competing interests.

## Supplemental Information

Document S1. Figure S1

Document S2. Article plus supplemental information
