# Supplementary material for: PAI1 regulating CHRNA1 contributes to primary focal hyperhidrosis: Clinical and experimental studies
Source: Mol Ther Nucleic Acids. 2025 May 16;36(2):102566. doi: 10.1016/j.omtn.2025.102566 (PMC12155562; doi:10.1016/j.omtn.2025.102566)
Supplement: Document S1. Figure S1 [file mmc1.pdf]

OMTN, Volume *36*

Supplemental information

PAI1 regulating CHRNA1 contributes to primary focal hyperhidrosis: Clinical and experimental studies

Ru-Jie Zheng, Nan-Long Lin, Meng-Long Zhang, Rui-Qin Qiu, Feng-Qiang Yu, Xu Li, and Jian-Bo Lin

Supplementary materials

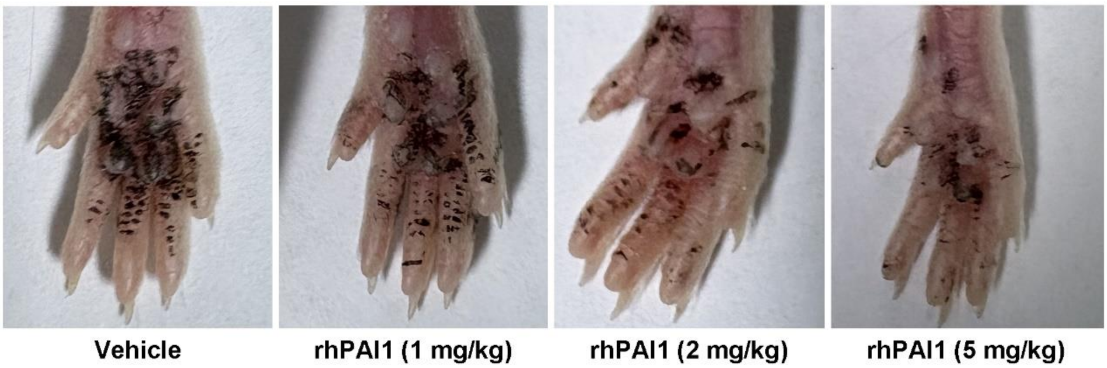

Figure S1. The representative images of the hyperhidrosis mice from each group.
